# Bone repair access of BoneCeramic™ in 5-mm defects: study on rat calvaria

**DOI:** 10.1590/1678-7757-2016-0531

**Published:** 2018-01-16

**Authors:** André Luis da Silva FABRIS, Leonardo Perez FAVERANI, Pedro Henrique Silva GOMES-FERREIRA, Tárik Ocon Braga POLO, Joel Ferreira SANTIAGO-JÚNIOR, Roberta OKAMOTO

**Affiliations:** 1 Univ. Estadual Paulista (UNESP) - Faculdade de Odontologia de Araçatuba, Departamento de Cirurgia e Clínica Integrada, Araçatuba, S P, Brasil.; 2 Universidade do Sagrado Coração, Departamento de Ciências da Saúde, Bauru, SP, Brasil.; 3 Univ. Estadual Paulista (UNESP) - Faculdade de Odontologia de Araçatuba, Departamento de Ciências Básicas, Araçatuba, S P, Brasil.

**Keywords:** Biocompatible materials, Bone regeneration, Immunohistochemistry

## Abstract

**Objective:**

The aim of this study was to evaluate the osteoconductive potential of BoneCeramic™ on bone healing in rat calvaria 5-mm defects.

**Material and Methods:**

A 5-mm calvaria bone defect was induced in three groups and the defect was not filled with biomaterial [Clot Group (CG)], autogenous bone (AG), or Bone Ceramic Group (BCG). Animals were euthanized after 14 or 28 days and the bone tissue within the central area of the bone defect was evaluated. Results were compared using ANOVA and Tukey test (p<0.05). Immunohistochemistry was performed using primary antibodies against osteocalcin, RUNX-2, TRAP, VEGF proteins, and 3-dimensional images of the defects in μCT were obtained to calculate bone mineral density (BMD).

**Results:**

In BCG, the defect was completely filled with biomaterial and new bone formation, which was statistically superior to that in the GC group, at both time-points (p<0.001 for 14 days; p=0.002 for 28 days). TRAP protein showed weak, RUNX-2 showed a greater immunolabeling when compared with other groups, VEGF showed moderate immunostaining, while osteocalcin was present at all time-points analyzed. The μCT images showed filling defect by BCG (BMD= 1337 HU at 28 days).

**Conclusion:**

Therefore, the biomaterial tested was found to be favorable to fill bone defects for the reporting period analyzed.

## Introduction

Functional recovery of the jaws using dental implants requires a minimum thickness and height of ridges^1^ ^,^ ^5^ ^,^ ^15^. Several factors culminate in the absence of alveolar bone, which then becomes a limiting factor for implant placement. This usually occurs in patients who have suffered dentoalveolar trauma, traumatic tooth extractions, dental congenital absence, or other diseases involving the maxilla and mandible^2^ ^,^ ^9^ ^,^ ^17^.

The current trend is to develop and use biomaterials that accelerate or at least allow normal and complete repair of bone defects, thus reducing postoperative failure rate^7^ ^,^ ^22^. The objective of tissue engineering is to generate an osteoconductive biomaterial that is conducive to bone healing or even superior to bone grafts^17^.

Synthetic bone substitutes with osteoconductive property, such as calcium phosphate-based ceramic biomaterials, have been investigated^10^ ^,^ ^11^ ^,^ ^20^. Recently, Miramond, et al.^14^ (2014) showed that isolated biphasic calcium phosphate has osteoinductive potential when used within subcutaneous tissue. The physical properties of biomaterials vary as *per* their surface area or the format (block or particle), porosity (dense, micro-, or macroporous) and crystallinity (crystalline or amorphous). On the other hand, chemical properties are related to the chemical composition of the material such as calcium/phosphate molar ratio, the level of elemental impurities, and ionic substitution in the atomic structure^24^.

Straumann^©^ BoneCeramic™ (BC) is a 100% synthetic bone substitute with morphology to stimulate the formation of vital bone. It has 90% porosity index, with interconnected pores with 100 to 500 microns diameter and is composed of biphasic calcium phosphate, with 60% hydroxyapatite (HA) and 40% β-tricalcium phosphate. The mechanical stability of the increased volume is maintained due to the slow resorption of HA, which prevents excessive resorption, according to the manufacturer instructions. Studies by Daculsi, et al.^4^ (2003), Cordaro, et al.^3^ (2008), Frenken, et al.^6^ (2010), Mardas, et al.^12^ (2011), and Wang, et al.^23^ (2014) showed that BC is a bone substitute with osteoconductive properties, favorable for repair of bone defects, with similar results for amount of mineralized bone when compared with inorganic bovine bone (Bio-Oss^®^). However, there was a greater amount of soft tissue component and different features of resorption for the BC group^3^, therefore, these authors have recommended further studies to confirm the results.

The aim of this study was to evaluate the osteoconductive potential of HA granules and β-tricalcium phosphate (BoneCeramic – Straumann^®^) in the repair of 5-mm defects in rat calvaria, using histometry, immunohistochemistry, and microtomographic analysis, at 14 and 28 days, compared with defect not filled with biomaterial (negative control) or autogenous bone graft (positive control).

We propose the null hypothesis that the bone area of newly formed bone defects filled with autogenous bone or BC will not show a statistical difference.

## Material and methods

### Experimental design

This research was approved by the Ethics Committee on Animal Experiments (process 2012/01097). A total of 56 adult (3–4-month-old) male rats (*Rattus norvegicus albinus*, Wistar), weighing approximately 200 to 300 g, were used. The animals were divided into three groups (n=8 each) and euthanized at one of the two time-points: 14 and 28 days after surgery. They were kept in cages and fed with balanced feed (NUVILAB, Curitiba, PR, Brazil) containing 1.4% Ca and 0.8% P and provided water *ad libitum* in the our institution.

A bone defect (5 mm) was induced in the calvaria of each animal as follows:

- Clot Group (CG, n=16): The bone defect was not filled with some biomaterial (the defect remain empty until clot stabilization);
- Autogenous Bone Group (AG, n=16): The 5-mm bone defect was filled with autogenous bone (collected from the calvaria of the same animal). After the defect was made, the bone was particulate and used to fill the defect;
- Bone Ceramic Group (BCG, n=24): The 5-mm bone defect was filled with alloplastic bone (Straumann). For this group, eight additional animals were operated upon and euthanized after 42 days to verify the long-term behavior of this group.

### Experimental surgery

The animals were subjected to preoperative 12-hour fasting and sedated by intramuscular administration with Coopazine^®^ (10 mg/kg xylazyne; Coopers Ltd., Brazil) and Vetaset^®^ (75 mg/kg ketamine hydrochloride; Saúde Animal Ltd., Fort Dodge, IA, USA). Trichotomy was carried out in the region of the calvaria, antisepsis with polyvinylpyrrolidone iodine degerming (PVPI 10% Riodeine degerming, Rioquímica, São José do Rio Preto, SP, Brazil), associated with topical PVPI (PVPI 10% Riodeine, Rioquímica, São Jose do Rio Preto, SP, Brazil) and affixed barren areas.

A U-shaped incision was made in the occipitofrontal sense, based toward the posterior region with a no. 15 blade (Feather Industries Ltd., Tokyo, Japan) mounted on a no. 3 scalpel handle (Hu-Friedy, Germany) and the total detachment of the flap with peeler Molt type (Hu-Friedy, Germany). Using a trephine drill aid (3i Implant Innovations, Inc., Palm Beach Gardens, USA) coupled in low rotation speed under abundant irrigation with sodium chloride solution 0.9% (Darrow, Rio de Janeiro, RJ, Brazil), a 5-mm surgical defect was made at the central portion of the calvaria involving the sagittal suture, maintaining the integrity of the dura^21^. According to the proposed treatments, the defects were not filled with biomaterial (n=16), autogenous bone (n=16), or synthetic bone – Straumann Bone Ceramic (n=24).

To finish the procedure, the soft tissues (including the periosteum) were carefully repositioned and sutured at the surface with a resorbable wire (polylactic acid – Vycril 4.0, Ethicon, Johnson Prod, São José dos Campos, SP, Brazil) and in deeper areas using monofilament plan (Nylon 5.0, Mononylon, Ethicon, Johnson Prod., São José dos Campos, SP, Brazil) with the most points externally interrupted.

During the immediate postoperative period, each animal received a single intramuscular dose of 0.2 mL penicillin G benzathine (small-size veterinary pentabiotic, Fort Dodge Saúde Animal Ltda., Campinas, SP, Brazil). The animals were kept in individual cages throughout the experiment with food and water supplied *ad libitum*.

The animals were postoperatively euthanized at 14 or 28 days with an overdose of anesthetic (thiopental sodium, 150 mg/kg). In the BCG group, eight animals were postoperatively euthanized at 42 days.

Calvaria of rats were removed and fixed in 10% formaldehyde solution for 48 hours, washed in running water for 24 hours, decalcified in 20% ethylenediaminetetraacetic acid for 6 weeks followed by alcohol dehydration, and cleared in xylene. Subsequently, the calvariae were longitudinally cut in half, separating the bone defects. The specimens obtained were embedded in paraffin and cut using a microtome into 6-μm-thick sections and placed on slides. Some slides were stained with hematoxylin and eosin (HE) while others were used for immunohistochemical analysis.

Prior to performing the analyses, samples were coded in such a way that only the supervisor was aware of the group corresponding to each slide. Subsequently, the examiner performed the analyses in a blinded manner.

### Histometric analysis

After staining the slides in HE (Merck & Co., Inc.), measurements were performed using a ×1.6 magnifying glass (LeicaR DMLB, Heerbrugg, Switzerland) and a lens with ×20 magnification (Leica Microsystems Aristoplan, Leitz, Benshein, Germany). These were coupled to an image-capturing camera (LeicaR DC 300F microsystems ltd, Heerbrugg, Switzerland) and connected to a computer with software to analyze the scanned images (Image J, Image Processing and Analysis Software, Ontario, Canada). These images were stored in JPEG format for subsequent analysis and projected onto the screen of a Samsung monitor (SyncMaster 3NE, 15 inches). Initially, the program was calibrated by the “set scale” tool, from the ruler image photographed under a microscope with ×1.6 magnification by the magnifying glass and ×20 by the lens. Thus, all images held according to the pattern of capture groups in the magnification of ×1.6 and ×20.

Hence, the images of the groups were analyzed using the Image J program. Using the “hands free” tools, the area of newly formed bone tissue was selected from the osteotomized bone stumps, as well as the area of connective tissue, biomaterial, and blood clot, while the program estimated the size in μm^2^.

### Statistical analysis

Statistical analysis was performed using Sigma Plot software 12.3 (San Jose, CA, USA) as *per* standards from previous studies^8^ ^,^ ^11^ ^,^ ^16^. The comparative analysis of biomaterials was performed using 2-criteria variance analysis, after having performed the test of normality (Kolmogorov-Smirnov) and the equal variance test. Specific analysis for BCG (14, 28, and 42 days) was performed using analysis of variance (ANOVA) to a criterion^25^ after having performed the normality test (Kolmogorov-Smirnov) and equal variance test. Analysis of the time periods was performed using paired t-test^8^ ^,^ ^16^ and the normality test (Shapiro-Wilk) was used. For all analyses, the Tukey test was used as a post-test. We adopted a significance level of p<0.05.

### Immunohistochemical analysis

The immunohistochemistry analysis included the following steps: inhibition of endogenous peroxidase activity with hydrogen peroxide, antigen retrieval with citrate buffer at 60°C for 20 minutes, and blocking of unspecific reactions with skim milk and bovine albumin during incubation of antibodies.

We used primary antibodies (dilution 1:100) against transcription factor 2 Runt-related (RUNX-2), tartrate-resistant acid phosphatase (TRAP), vascular endothelial growth factor (VEGF), and osteocalcin (OC) (Santa Cruz Biotechnology), biotinylated secondary antibody, with 1:200 dilution (Pierce Biotechnology), the Streptavidin Biotin amplifier (Dako), and diaminobenzidine (Dako) as a chromogen. At the end of the reactions, counter-staining with Harris hematoxylin was performed. Negative controls were used (by omission of the primary antibodies) to avoid false positives.

For the analysis, an optical microscope with increased objective ×40 Leica Microsystems Aristoplan (Leitz, Benshein, Germany) coupled to an image-capture camera (Leica DFC 300FX, Leica Microsystems, Heerbrugg, Switzerland) and connected to a microcomputer with a software capable of analyzing scanned images (Leica Camera Software Box, Leica Imaging Manager -IM50 Demo Software) was used. A total of three images were obtained from each strip for analysis: the two edges and the center of the defect. For comparison of immunostaining intensity between the experimental groups, scores were created in which immunostaining was classified on a scale of 0 to 3 (absent – 0, mild – 1, moderate – 2, and intense – 3). The immunostaining was represented by average percentage of labeling for each protein evaluated. Thus, score 1 showed about 25% of immunolabeling; score 2: 50%, and score 3: 75% of immunolabeling. These patterns are based on the studies of Pedrosa, et al.^18^ (2009) and Ramalho-Ferreira, et al.^19^ (2016).

### Microtomographic analysis

After euthanasia (at 14 and 28 days for all groups and after 42 days for the additional group BC) and removal of the calvaria, they were fixed in paraformaldehyde and, prior to laboratory processing to obtain the histological slides, two samples of each group and time period were subjected to the evaluation through a computer-assisted microtomograph (μCT; Bruker micro CT, Skyscan, S), using sections of 9 μm in thickness (60 Kv and 165 ***μ***A), with aluminum (0.5 mm) filter and 0.6-mm rotation step, pixel size of 9.92 *μm a*nd acquisition time of 23 minutes. The images obtained by the projection of X-rays in the samples were stored and reconstituted determining the area of interest by NRecon software (SkyScan 2011, Version 1.6.6.0), with smoothing of 1 artifact rings correction of 5, beam hardening correction of 20% and image conversion varied from 0.0 to 0.15. The pattern of new bone formation was evaluated by 3-dimensional images obtained by Datavier and CTvox software (Skyscan, Be). Bone mineral density (BMD) was assessed by the CTan software (Skycan, S) in Hounsfield Units (HU), by measuring the mineral density at the central area of the defect in the different experimental periods (14, 28, and 42 days).

## Results

### Histological/histometric analysis

**Figure 1:**
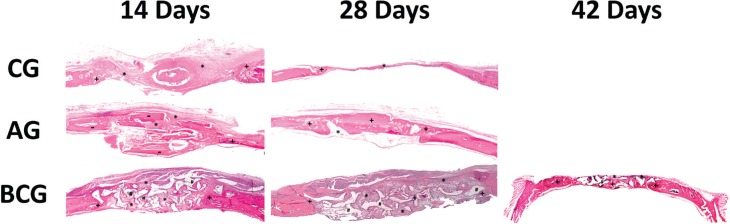
Histological images of experimental groups (CG at 14 days and CG at 28 days; AG at 14 days and AG at 28 days; BCG at 14 days, 28 days, 42 days. (1.6x magnification – Loupe). Different signs were used to identify the type of tissue: *=Connective tissue; += Neoformed bone; #=BoneCeramic particles; -=Autograft particles

**Figure 2:**
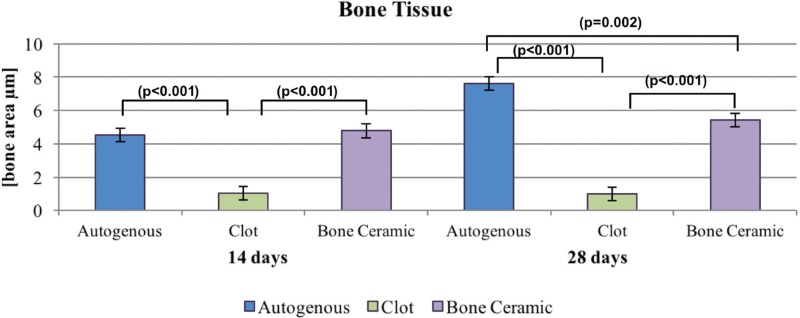
Neoformed bone tissue at 14 and 28 days

**Figure 3:**
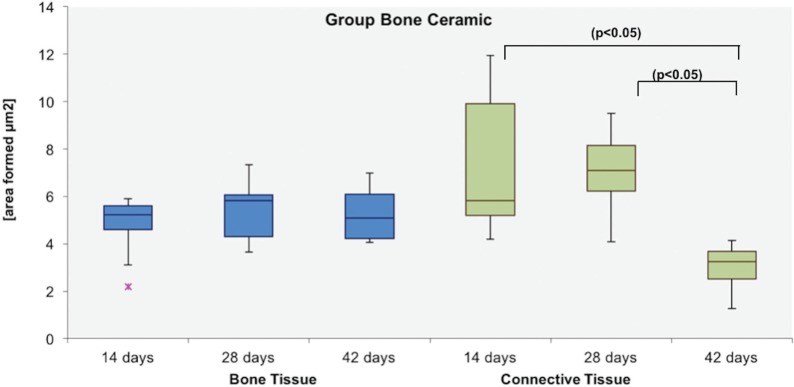
Graphic box-plot showing BCG. Bone and connective tissue at 14, 28 and 42 days

The site of the bone defect originally treated with BC was filled by a conglomerate of biomaterial and newly formed bone. BC remnants were visible and were in close contact with the bone tissue. Furthermore, we observed similar results in the GA group (Figure 1). The Tukey post-test indicated that, at 14 days, the autogenous bone formed more bone compared with the clot (p<0.001), and similar results were observed in the BC versus (vs.) clot (p<0.001) groups. At 28 days, the autologous bone showed a greater area of bone formation when compared with the clot (p<0.001) and the BC group (p=0.002). Thus, BCG was more favorable for the formation of bone tissue when compared with the clot (p<0.001). Clot was the most unfavorable group, as shown in Figure 2. Regarding the time for each biomaterial, it was observed that there was an increase in autogenous bone tissue formation from 14 to 28 days (p=0.002), but the other groups showed no statistically significant difference for the clot (p=0.845) and BC groups (p=0.511). A specific analysis of the bone ceramic group indicated that there was no statistically significant difference in bone formation at 14 vs. 28 days (p=0.511), 14 vs. 42 days (p=0.319), and 28 vs. 42 days (p=1.000) (Figure 3).

Defects that were not filled with biomaterial (GC) had a thin connective tissue closing the bone defect. The BC group may have favored the formation of bone, which was observed in the samples at 14, 28, and 42 days, and neogenesis was similar in the three periods. As expected, no substantial bone formation was observed in the group clot defects, while the particulate autogenous bone group resulted in the closure of the original defects (Figure 1).

**Figure 4:**
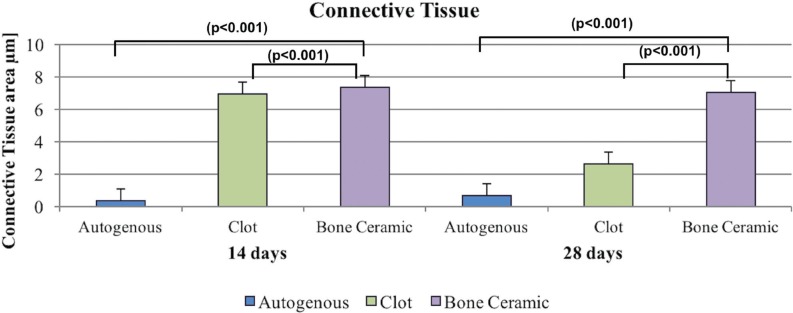
Neoformed connective tissue at 14 and 28 days

At 14 days, BCG showed increased formation of connective tissue compared with the autologous group (p<0.001) and the clot group showed increased formation of connective tissue compared with the autologous group (p<0.001). However, there was no significant difference in the comparison of BCG and clot (p=0922) groups, power test α=1.0. At 28 days, BCG showed the highest formation of connective tissue compared with the autologous and clot groups (p<0.001) (Figure 4).

### Immunohistochemistry

**Figure 5:**
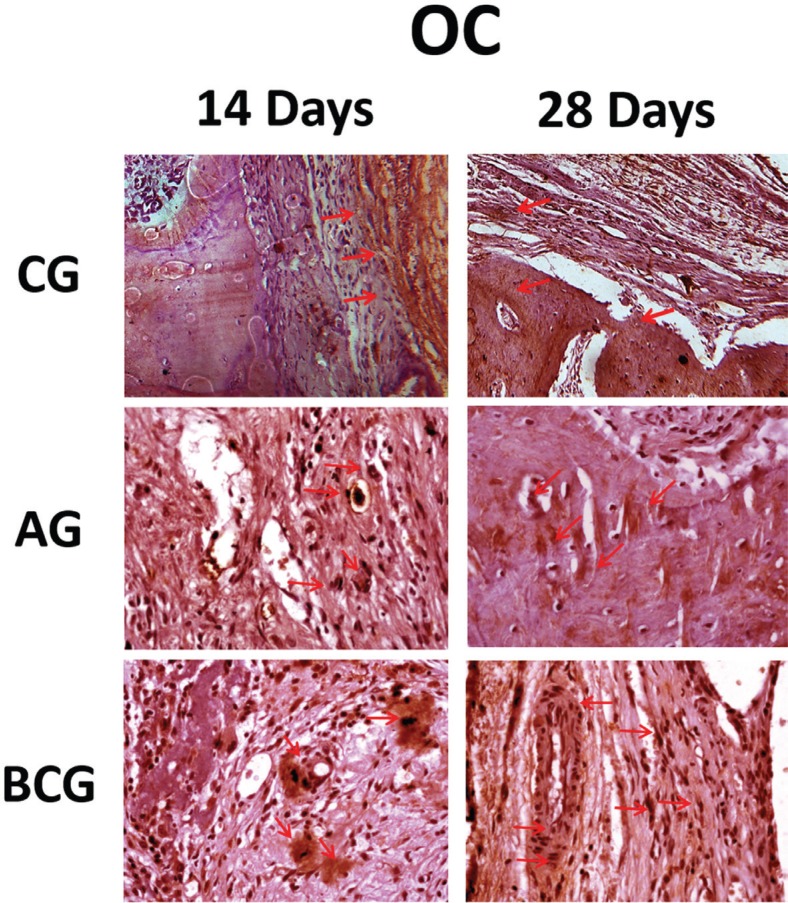
Osteocalcin immunostaining (OC) at 14 days for the experimental groups (CG: light; AG: moderate; BCG: moderate). At 28 days (CG: light; AG: moderate; BCG: moderate). Immunolabeling indicated by red arrows

In the analysis of immunoblots, positive markings were observed on the osteoblasts and extracellular matrix of the three evaluate experimental groups. Specifically at 28 days, in areas with bone formation, osteocalcin labeling was observed near to the mineralized bone matrix, showing the greater maturity of these specimens. Light immunostaining was observed for the control group at 14 and 28 days. For AG and BC groups, in both periods, immunostaining was moderate (Figure 5), but it is important to note that the AG has a great quantity of positive labeling in the mineralized bone matrix.

**Figure 6:**
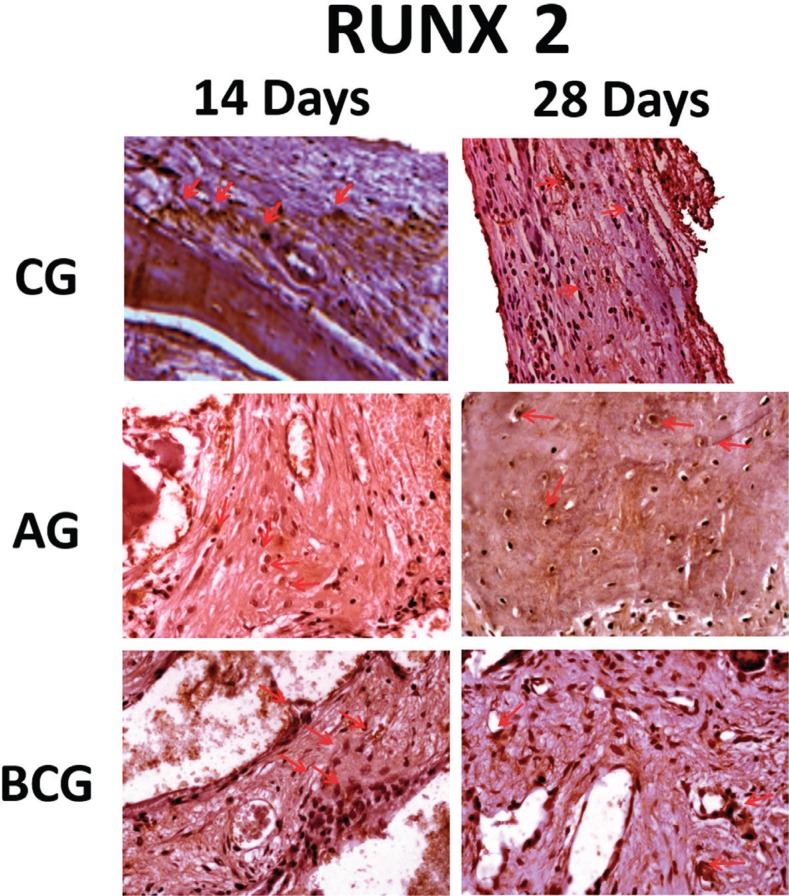
RUNX-2 immunostaining at 14 days for the experimental groups (CG: light; AG: moderate; BCG: moderate) and at 28 days (CG: light; AG: moderate; BCG: light). Immunolabeling indicated by red arrows

RUNX-2: In the three experimental groups, we observed positive staining of this transcription factor in very young pre-osteoblasts, osteoblasts and in some cases, osteocytes, in particular in the AG and BC groups. However, at 28 days, particularly in the clot and BC groups, there was a drop in positive staining of this factor in the cells of osteoblast lineage. This protein was lightly expressed in BCG (14 and 28 days), whereas in AG it was moderately expressed at 14 and 28 days. As for the BCG at 14 days it was observed as moderate, but at 28 days, there was a decrease in the immunostaining (light) (Figure 6).

**Figure 7:**
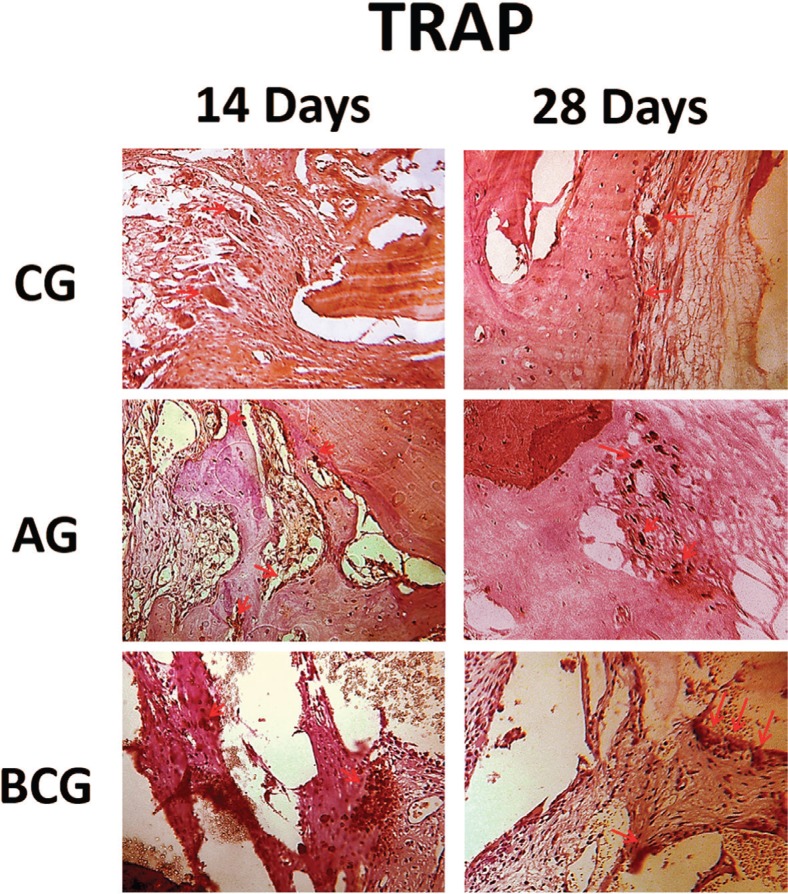
TRAP immunostaining at 14 days for the experimental groups (CG: light; AG: moderate; BCG: light) and at 28 days (CG: light; AG: light; BCG: moderate). Immunolabeling indicated by red arrows

TRAP: At 14 and 28 days for the control group, the immunostaining of this protein was light. For AG, it was moderately marked at 14 days and there was decreased expression at 28 days (light). This was in contrast to BCG, which showed slight immunostaining at 14 days and moderate at 28 days (Figure 7).

**Figure 8:**
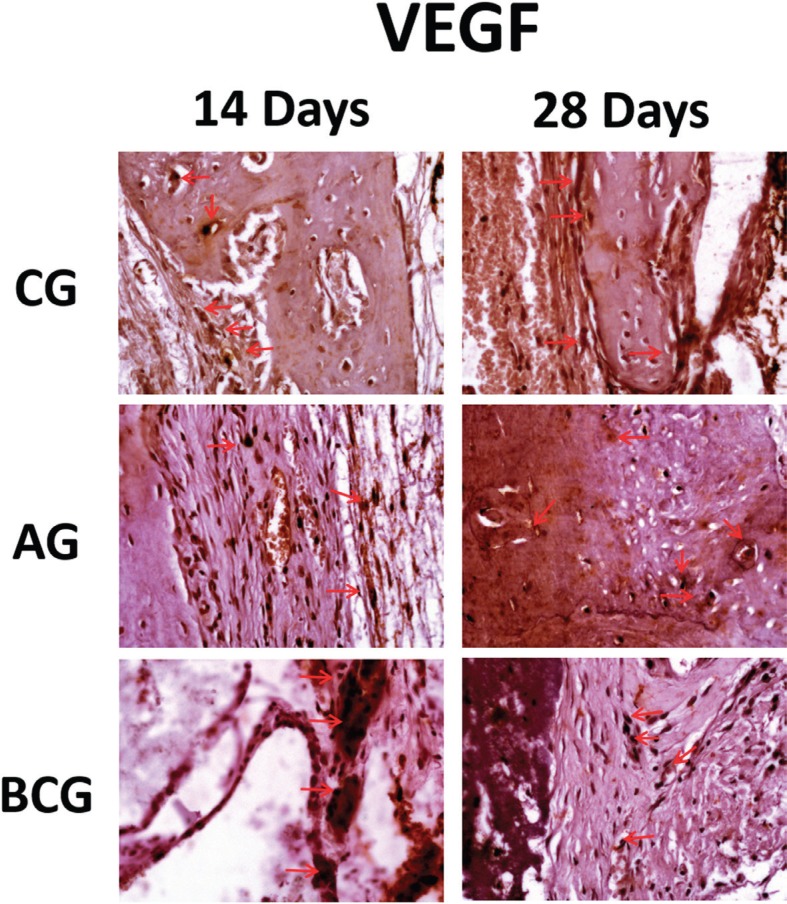
VEGF immunostaining at 14 days for the experimental groups (CG: moderate; AG: light; BCG: moderate) and at 28 days (CG: moderate; AG: moderate; BCG: moderate). Immunolabeling indicated by red arrows

VEGF: An important positive staining for this growth factor was observed in the three experimental groups, particularly the CG and BC groups, for which this protein was moderately expressed in both periods (14 and 28 days) analyzed. On the other hand, AG was expressed lightly at 14 days and moderate (especially close to mineralized matrix) at 28 days (Figure 8).

### Microtomography

**Figure 9:**
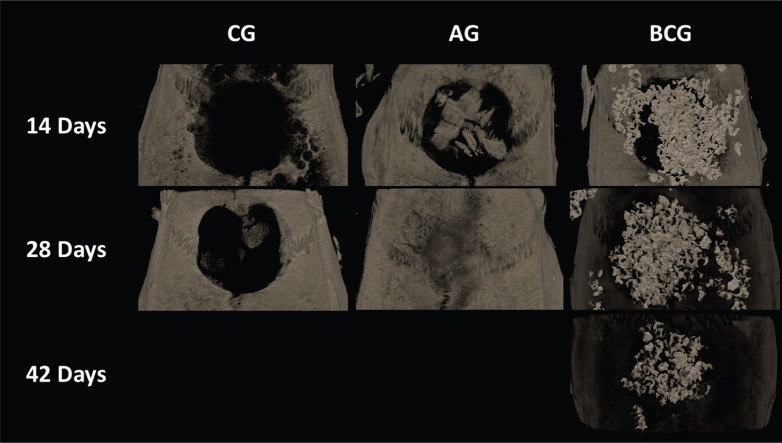
Images obtained by μCT of CG, AG, and BCG (14, 28 and 42 days). Bone neoformation was observed with total filling of defect at 28 days in GA. BCG promoted filling of defect with the biomaterial until the 42-day period

**Figure 10:**
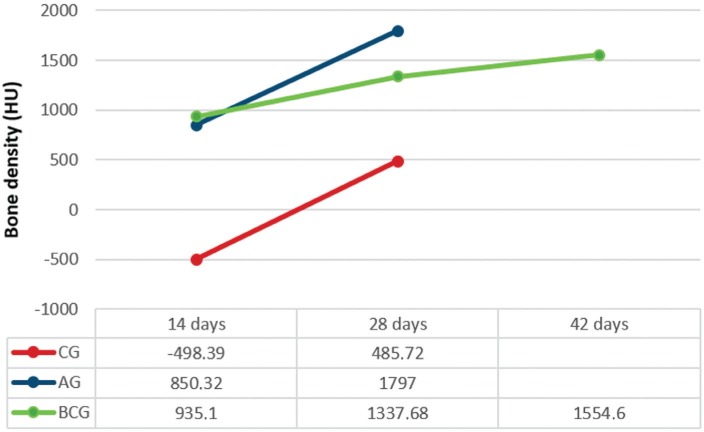
BMD average data obtained by μCT of CG, AG, and BCG (14, 28 and 42 days)

Images obtained by μCT showed the defect filled by BC (14, 28, and 42 days) and AG (14 and 28 days). The AG group showed full compatibility bone formation after 28 days; however, the defect was not fully filled in the CG as expected (Figure 9). The average BMD for the CG, AG, and BCG was 498, 850, and 935 HU at 14 days and 485, 1797, and 1337 HU at 28 days, respectively. Moreover, the BMD for BCG was 1554 HU at 42 days (Figure 10).

## Discussion

We rejected the null hypothesis that the newly formed bone area within the bone defect filled with autogenous bone or BC would not show a statistical difference, considering that for all analyzed periods the autologous bone showed a greater extent of bone formation compared with the other biomaterials (p<0.05). However, BC was satisfactorily responsible for filling bone defects, when compared with the control group (p<0.05), with mean BMD values similar to GA.

The results obtained in our study indicate that the tested biomaterial (BCG) with the clot was favorable for recovery of the bone defect compared with filling with the clot alone. However, even though the bone quantity observed in BCG was lesser in extent than the autograft, this bone quantity was maintained in all of the evaluated periods, being important to fill the calvaria defect. This was probably due to HA (60%) in the composition of BoneCeramic^®^, which has very low absorption. Thus, the material remained at the insertion site, filling the defect until 42 days, in contrast to the autogenous bone graft, which closed the defect with mineralized bone at 28 days.

As BCG showed a small amount of newly formed bone with full closure of the defect at 28 days when compared with the positive control group, it was noteworthy to determine BC results at a later time-point. Considering that this group presented important labeling of RUNX2, that is, immunoblotting referring to pre-osteoblastic cells that may be indirectly related to a possible tendency of late bone formation. Therefore, the 42-day BCG analysis was conducted.

Similarly as performed in our study, Takuti, et al.^21^ (2014) also evaluated the bone repair of BC in 5-mm size defects, but in defects with 8 mm of diameter and rabbits' calvaria. These authors found greater percentage of newly bone formed to BC groups (78.06%) when compared with xenograft (Bio-Oss: 57.76%; Endobon: 58.42%). A surprising statistical interaction with control group (clot: 77.8%) was observed. Furthermore, these data were not supported by representative histological images, which did not seem to show the same results observed by histometric analysis. For this reason, our study was important to show some differences to BC behavior in a lower defect (5 mm) and another animal experimental model (rats).

Regarding osteoconductive property of the biomaterials, Luvizuto, et al.^11^ (2011) employed β-tricalcium phosphate (βTCP 100%, Cerasorb^®^) and other biomaterials (polylactic and polyglycolic acid gel and calcium phosphate cement) for filling 5-mm bone defects in the calvaria of rats at day 45 post-surgery. These biomaterials were used alone or supplemented with 5 μg bone morphogenetic protein (BMP-2) and compared with defects filled with autogenous bone. The authors observed that TCP alone promoted bone formation more efficiently compared with autografts and that BMP-2 supplementation was not required for filling bone defects. TCP was resorbed and allowed formation of mature bone tissue, closing the surgical defect. Thus, the results of these studies show that the presence of HA, with low absorption filled the bone defect at the last time-point in this study, with a lower rate of bone formation. Clinically, Daculsi, et al.^4^ (1999) observed a resorption rate of about 50% with BC used as a graft in the maxillary sinus membrane at 1 year post-operation, confirming the slow resorption of the material.

This is not to say that the preservation of the material, without full closure of the defect with neoformed bone is unfavorable for bone repair; however, only one may ask about the quality of this bone to support dental implants, first regarding the primary stability of the implant and then regarding osseointegration. Results obtained with microtomographic evaluation were encouraging in this context, very similar to the BMD values of autogenous bone. Marx, et al.^13^ (2014) reported that BMD of 737 HU was obtained after the reconstruction of critical defects with rh-BMP2, which is satisfactory for primary stabilization of dental implants. This indicates that BC is favorable, considering that BMD at 42 days in this study was 1554 H.

Previous studies that analyzed BC and inorganic bovine bone (Bio-Oss^®^) in surgeries to gain height in the maxillary sinus region or in the completion of post-exodontic alveoli show no statistically significant differences regarding the amount of mineralized bone between the tested biomaterials^3^ ^,^ ^21^. However, these studies did not verify the stability of dental implants after implantation, which is the main objective of oral rehabilitation. Frenken, et al.^6^ (2010) conducted a study to evaluate the quality and quantity of bone formed in the sinus floor elevation with BoneCeramic^©^, 6 months post-surgery, showing that the vertical height achieved was maintained. The implants seemed osseointegrated even 3 months after repair. Therefore, further studies to investigate this situation should be performed, evaluating the stability of the implants at different times by resonance frequency analysis, since BC at 28 and 42 days in this study showed adequate BMD. Thus, filling the bone defect with BC showed long-term stability of the osseointegrated implant after installing the prosthesis.

In immunohistochemical results, osteoblast differentiation, indicated by RUNX-2 expression, showed low osteoblastic activity in CG at both time-points (14 and 28 days), confirmed by histological analysis, in which the defects were filled by connective tissue with low presence of osteoblastic lineage cells. In AG, this protein was moderately marked specially in osteocytes at 14 and 28 days, which underlines the longevity of bone autograft formation. Unlike the defects filled with BoneCeramic^®^, where the expression of RUNX-2 was decreased at 28 days, but was moderate at 14 days, and was still present after 28 days, even if lightly. Thus, although the osteoconductive potential of this material is known, we suggest that its presence within the bone defect may lead to an osteoblast differentiation response, as evidenced by the expression of RUNX-2 transcription factor. Miramond, et al.^14^ (2014) evaluated the 2-phase graft hydroxyapatite (HA)/tricalcium phosphate (TCP) in rat subcutaneous tissue and confirmed the osteoinductive potential of these biomaterials. Therefore, further studies should be conducted to quantify this protein through molecular analysis using real-time polymerase chain reaction (RT-PCR), to better understand these findings.

In a consequence for RUNX-2 labeling, and osteoblastic differentiation, OC labeling showed that newly formed bone mineralization after preparation of the bone defects was similar for the biomaterials used (AG and BCG), both at 14 and 28 days, indicated by moderate immunostaining of the OC protein. Defects left unfilled with particulate biomaterials (GC) had mild levels of this protein. Thus, OC, the most abundant non-collagen protein of the bone matrix, expressed during bone mineralization also represents marking cells of osteoblastic lineage, including osteoblasts and osteocytes, and it showed no difference in expression between the biomaterials tested. Thus, the comparison between BCG and CG, by OC expression, suggests that Ceramic Bone^®^ was favorable for the filling of the defect created in rat calvaria.

Indirectly RUNX-2 and OC labeling representing the osteoblast activity give support to evaluate the osteoconduction property of the biomaterials filling the defect. In addition, angiogenesis after tissue damage, marked by VEGF protein expression, showed in this *in vivo* investigation that the blood supply, in particular to combat local oxygen deficit was moderate in both periods analyzed for the CG and BC groups. Particularly in BCG, the presence of VEGF characterizes its known osteoconductive potential. Since autologous bone already has viable cells for repair, including endothelial cells, VEGF was lightly immunostained at 14 days and the intensity increased at 28 days.

Osteoclast activity observed by TRAP expression demonstrated that autologous bone resorption decreased, whereas in BCG, resorption increased over time. This indicated that the biomaterial remained reabsorbed, and therefore delayed bone healing. Future studies with longer analysis time should be performed to investigate whether the reabsorption remains in this group, with more filling of the defect with mature bone.

## Conclusion

Therefore, within the limits of this *in vivo* study, we may conclude that the tested biomaterial (BC) was favorable to fill bone defects until the end of the reporting period analyzed.
